
JOURNAL INFORMATION
==============================
NLM Title Abbreviation: ScientificWorldJournal
Journal ID: ScientificWorldJournal
NLM Journal ID: 101131163
Journal ID: TSWJ

The Scientific World Journal

ISSN: 2356-6140
EISSN: 1537-744X
Publisher: Wiley

ARTICLE INFORMATION
==============================
PMCID: PMC5823230
PMID: 19578710
DOI: 10.1100/tsw.2009.78
Article ID: 542702
Article version: 1
Subjects: Editorial

The Rise of Environmental Analytical Chemistry as an Interdisciplinary Activity

Brown Richard J. C.
Analytical Science Team, National Physical Laboratory, Teddington, Middlesex, UK
Publication date: 2009
Electronic publication date: 2009 Jul 1
Volume: 9
First page: 536
Last page: 538
Received 2009 May 21; Revised 2009 Jun 10; Accepted 2009 Jun 12
Copyright: Copyright © 2009 Richard J. C. Brown.
Copyright year: 2009
License: This is an open access article distributed under the Creative Commons Attribution License, which permits unrestricted use, distribution, and reproduction in any medium, provided the original work is properly cited.
License URL: https://creativecommons.org/licenses/by/3.0/

==============================
Editorial
*TheScientificWorldJOURNAL* (2009) 9, 536–538
ISSN 1537-744X; DOI 10.1100/tsw.2009.78

TheScientificWorldJOURNAL
www.thescientificworld.com

### The Rise of Environmental Analytical Chemistry as an Interdisciplinary Activity‡

Richard J.C. Brown
*Analytical Science Team, National Physical Laboratory, Teddington, Middlesex, U.K.*

**E-mail**: richard.brown@npl.co.uk

*Received May 21, 2009; Revised June 10, 2009; Accepted June 12, 2009; Published July 1, 2009*

---

Modern scientific endeavour is increasingly delivered within an interdisciplinary framework (or multi- or cross-disciplinary, if one prefers[1]). In part, this is due to the need for collaboration between subject areas to address the current topics of interest, and partly because of the blurring of traditional subject areas and disciplines as new areas of research emerge which do not fit neatly into existing categories (for instance, nanotechnology).

Against this background, it is clear that environmental chemistry (the study of the sources, transport, effects, reactions, and fates of chemical and biochemical species in air, soil, and water environments and, importantly, human effects on these) and analytical chemistry (the study of the chemical composition of mixtures[2]) are, and always have been, closely related. Environmental chemistry relies on analytical chemistry to deliver its aims. Equally, much of analytical chemistry is driven by the requirements of environmental chemistry. A virtuous circle exists in this relationship, where improvements in analytical chemistry (such as techniques to address new analytes or provide lower detection limits[3]) allow advances in environmental chemistry that drive further improvements in analytical chemistry, and so on. Whilst other interdisciplinary input is, of course, present (from toxicologists, epidemiologists and atmospheric chemists, to name but a subset[4]), "analytical environmental chemistry" – the application of analytical chemistry to environmental chemistry problems – is clearly a very important factor. This is a long-standing example of an interdisciplinary approach to scientific research where value is added by the close cooperation of different disciplines.

The recent proliferation of activities considered by those delivering them as "analytical environmental", rather than simply "analytical chemistry" or "environmental chemistry", can be gauged from a review of the number of peer-reviewed publications in these areas using widely available databases of published literature. The total peer-reviewed output in the chemistry area has increased by almost 250% since 1992; therefore, one would expect publication numbers in most subject areas to rise along similar lines if they maintained a consistent profile within the scientific community during this period. Fig. 1 shows the relative change in publication frequency of the three areas relevant to this discussion when compared to the overall increase in publication frequency in the chemistry area (represented by the 0% line). The number of publications in the "analytical chemistry" and "environmental chemistry" subject areas has shown a similar rise in publication numbers to that seen overall since 1992 (hence, a change in relative publication frequencies of around 0%). Conversely, the number of papers using both the keywords "analytical" and "environmental" has outstripped the increase in overall publication numbers by approximately 70% over the same period.

---

‡ The views expressed in this article are those of the author and do not necessarily represent the views of the National Physical Laboratory or NPL Management Limited.

©2009 with author.
Published by TheScientificWorld; www.thescientificworld.com

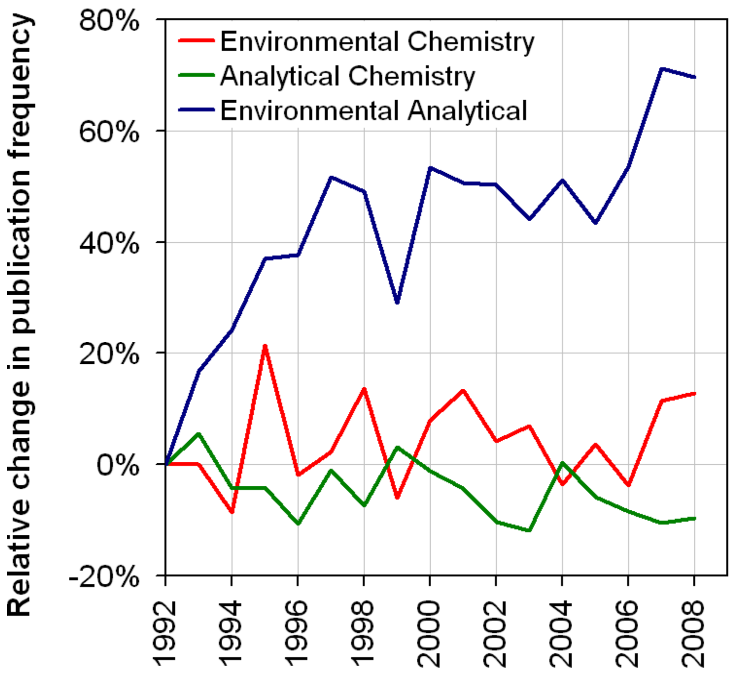

**FIGURE 1.** The relative change in frequency of publications with the keywords "environmental chemistry" and "analytical chemistry", and those publications using both the keywords "analytical" and "environmental", compared to the overall increase in publication frequency in the chemistry area (represented by the 0% line), since 1992.

### Scope of TSWJ's Analytical and Environmental Chemistry Domains

TheScientificWorldJOURNAL's (TSWJ's) innovative domain structure provides a framework for disseminating research of relevance to a number of subject areas. Moreover, in my role as Principal Editor of both the Analytical and Environmental Chemistry domains, I am in a good position to oversee the synergy between these two areas and their relevance to the rest of TSWJ's output.

Therefore, I would like to encourage submissions to TSWJ's Analytical Chemistry and Environmental Chemistry Domains, and also suggestions for special issues in these areas. The wide scope of TSWJ means that all contributions of new data, methods, case studies, and instrumentation, or new interpretations and developments of existing data, case studies, methods, and instrumentation, relating to analytical and/or environmental chemistry, are welcome and will be considered equally. Offers of review articles on relevant topics are also very welcome (but should be agreed with the Principal Editor before commencing work).

I especially invite studies that are interdisciplinary in nature (not just between the Analytical and Environmental Chemistry areas – see the full list of TSWJ domains[5]), as TSWJ provides the platform to ensure that such work reaches a wide and appropriate audience.

TSWJ maintains an Impact Factor of approximately 2.5 – highly competitive for a journal publishing in the Analytical and Environmental Chemistry areas.

### REFERENCES

1. http://en.wikipedia.org/wiki/Interdisciplinary (Accessed May 2009)
2. Brown, R.J.C. (2008) Quantities and units in analytical chemistry. *Int. J. Environ. Anal. Chem.* **88,** 681–687. DOI: 10.1080/03067310801899722

3. Brown, R.J.C. (2008) The use and abuse of limits of detection in environmental analytical chemistry. *TheScientificWorldJOURNAL* **8,** 796–801. DOI: 10.1100/tsw.2008.107
4. Garrigues, P. (2005) Environmental chemistry: the ultimate challenge in analytical chemistry. *Anal. Bioanal. Chem.* **381,** 3-4. DOI 10.1007/s00216-004-2931-4
5. http://www.thescientificworld.co.uk/TSW/DomainsAndClusters/alldomains.asp?menuid=17&jid=141&HL=5

**This article should be cited as follows:**

Brown, R.J.C. (2009) The rise of environmental analytical chemistry as an interdisciplinary activity. *TheScientificWorldJOURNAL* **9**, 536–538. DOI 10.1100/tsw.2009.78.
